# Effect of Planting Date and Cultivar Maturity in Edamame Quality and Harvest Window

**DOI:** 10.3389/fpls.2020.585856

**Published:** 2021-01-18

**Authors:** David Moseley, Marcos Paulo da Silva, Leandro Mozzoni, Moldir Orazaly, Liliana Florez-Palacios, Andrea Acuña, Chengjun Wu, Pengyin Chen

**Affiliations:** ^1^LSU AgCenter, Alexandria, LA, United States; ^2^Department of Crop, Soil, and Environmental Sciences, University of Arkansas, Fayetteville, AR, United States; ^3^Division of Plant Sciences, University of Missouri, Columbia, MO, United States

**Keywords:** edamame, quality, harvest date, planting date, color, pod weight

## Abstract

Edamame is a food-grade soybean [*Glycine max* (L.) Merr.] that is harvested immature between the R6 and R7 reproductive stages. To be labeled as a premium product, the edamame market demands large pod size and intense green color. A staggered harvest season is critical for the commercial industry to post-harvest process the crop in a timely manner. Currently, there is little information to assist in predicting the optimum time to harvest edamame when the pods are at their collective largest size and greenest color. The objectives of this study were to assess the impact of cultivar, planting date, and harvest date on edamame color, pod weight, and a newly minted Edamame Harvest Quality Index combining both aforementioned factors. And to predict edamame harvest quality based on phenological stages, thermal units, and planting dates. We observed that pod color and weight depended on the cultivar, planting date, and harvest date combination. Our results also indicated that edamame quality is increased with delayed planting dates and that quality was dependent on harvest date with a quadratic negative response to delaying harvest. Maximum quality depended on cultivar and planting and harvest dates, but it remained stable for an interval of 18–27 days around the peak. Finally, we observed that the number of days between R1 and harvest was consistently identified as a key factor driving edamame quality by both stepwise regression and neural network analysis. These research results will help define a planting and harvest strategy for edamame production in Arkansas and the United States Mid-South.

## Introduction

Edamame (vegetable soybean) is a food-grade soybean [*Glycine max* (L.) Merr.], which is harvested immature between the reproductive stages of R6 and R7, when the beans fill 80–90% of the pod (Konovsky et al., 1994; Shanmugasundaram and Yan, 2004). As a vegetable product, the appearance of the pod and bean must be acceptable for end consumers. The main physical attributes of edamame include large seed weight (>30 g per 100 seeds) and large and green crescent shaped pods with two or three seeds (Mentreddy et al., 2002; Shanmugasundaram and Yan, 2004). Production of edamame in the United States is thought to have started in the 1950s including home gardens and food processors. Demand for edamame in the United States has seen a dramatic increase since the early 2000s (Mentreddy et al., 2002). Nuss (2013) reported that between 22,600 and 27,000 Mg of edamame per year was consumed in the United States, estimated to be a $175–$200 million market. The United States is one of the top soybean-producing countries; therefore, soybean growers have the potential to produce edamame competitively, since commodity soybean and edamame share requirements of photoperiod sensitivity, fertilization practices, disease management, and irrigation techniques (Nuss, 2013; Ross, 2013; Ogles et al., 2016).

Soybean development and maturation are divided into vegetative and reproductive physiological stages (Fehr et al., 1971). Reproductive stages are characterized by blooming (R1 and R2), pod development (R3 and R4), seed filling (R5 and R6), and plant maturity (R7 and R8) stages (Fehr et al., 1971). Pod and seed appearance and seed composition change during these reproductive stages. Previous work from our research team, working on food-grade soybean cultivars including edamame materials, demonstrated that protein initially decreases for 3–5 weeks after flowering but then begins to accumulate, contrarily to oil that is accumulated steadily during the early reproductive stages (Saldivar et al., 2011). Also, starch and sucrose contents steadily decrease with seed development, while oligosaccharides remain low in seed until 3 weeks prior to R8 stage (Saldivar et al., 2011). In addition, Xu et al. (2016), working on two edamame cultivars, reported that seed weight peaks at the R6 stage, observed a continuous decrease in seed green color from R5 to R8 stage, and confirmed the report by Saldivar et al. (2011) on seed protein, oil, and carbohydrate accumulation patterns. Such drastic changes in soybean seed composition with stage of development highlight the importance of a timely harvest of edamame to ensure both maximum seed size and an optimal seed composition.

Edamame under commercial production is typically harvested using a modified green bean picker. To spread out crop risks and to even the flow of materials entering post-harvest processing facilities, the edamame crop is typically stagger-planted through various dates and maturity-group combinations. Nolen et al. (2016) reported that techniques such as these can extend the harvesting season to several months and that a staggered harvest is critical due to the short window a cultivar will have acceptable pod size and color. It has been reported that the range from reproductive stages R5.8–R7.0 can be 18–20 days (Purcell et al., 2014); however, Nolen et al. (2016) suggested that the harvest window for an acceptable edamame product can be less than 18 days.

Soybeans will mature faster as the nights become longer (Garner and Allard, 1920). Garner and Allard (1920) added that photoperiodism is a major factor in soybean yield. Johnson et al. (1960) indicated photoperiodism can affect later stages of reproductive development, not just triggering flowering. In addition, some soybean cultivars are less sensitive than others to delayed planting and changes in photoperiod (Johnson et al., 1960), whereas very early cultivars [maturity group (MG) 00 and 0] have been reported not to be sensitive (Polson, 1972). In addition, as the relative maturity increases, the soybean reproductive growth stages become increasingly more sensitive to long nights (Johnson et al., 1960; Major et al., 1975b).

The ability to predict the harvest date of many horticulture crops is based on accumulated thermal units (Tu) above a crop-specific base temperature throughout the crop’s growing season (Oliver and Annandale, 1998; Miller et al., 2001). The base temperature below which growth and development of soybean stop is 7°C (Boote et al., 1998). Previous research has suggested that it is possible to use temperature in correlation with growth (Major et al., 1975b), but it has also been reported that predicting soybean growth stages using thermal units may be no more accurate than using calendar days (Major et al., 1975a). Therefore, the objectives of this study were to first assess the impact of planting date and harvest date on edamame pod color and pod weight on three edamame cultivars of contrasting maturity and growth habit; second, to identify the effect of planting date and harvest date on a newly defined Edamame Harvest Quality Index (EHQI) for each of the three aforementioned cultivars; and, third, to predict edamame harvest quality based on phenological stages, thermal units, and planting dates using Stepwise Regression and Artificial Neural Network Analysis.

## Materials and Methods

### Field Experimental Design

The experiment was designed as a split-split plot with three replications. The whole plot was planting date (three levels), the split-plot was edamame cultivar (“8080,” “R08-4002,” and “R09-345”), and the split-split plot was harvest date (eight levels) nested within planting date by cultivar. Harvest was initiated when a cultivar within a planted date reached R5.8 stage (Fehr et al., 1971) on the plot assigned for the first Harvest Date and continued approximately every 5 days on each of the subsequent Harvest Date plots. Harvesting was discontinued when the crop reached R7 (yellowing of pods); therefore, not all harvest date plots were used for every cultivar and planting date, as the total number of harvests depended on speed of crop progression to R7 stage. It is noteworthy that because the three cultivars in this study represent different MGs and because of the variation in photoperiod and temperature across planting dates, a given cultivar could be at different physiological stages across planting dates even if harvested the same number of days after R5.8.

Of the cultivars used, 8080 was an indeterminate MG3 cultivar, whereas R08-4002 and R09-345 were determinate MG5 and MG6 breeding cultivars, respectively. The experiment was grown over 2 years (2014 and 2015) in two locations, the Arkansas Agricultural Research and Extension Center (AAREC) in Fayetteville, AR, United States and the Vegetable Research Station in Kibler, AR, United States. Soils of the former are silt loam (Johnsburg Series; fine-silty, mixed, active, mesic Aquic Fragiudults), while for the latter were very fine sandy loam (Roxana Series; coarse-silty, mixed, superactive, nonacid, thermic Typic Udifluvents) (Soil Survey Staff, 2017). The three planting dates were mid to late May (PD1), mid to late June (PD2), and mid of July (PD3), representing planting dates typically used for edamame production in Arkansas. Each plot consisted of four rows 10.7 m long and 0.91 m wide. The seeding rate was 33 seeds per meter row, resulting in approximately 16 seeds per meter row at emergence. The plots were managed using standard agricultural practices for irrigation, fertilizer, and pesticides. At harvest, a total of 100 pods were randomly picked by hand throughout the canopy within the middle two rows of each four-row plot. The pods were immediately sealed in plastic bags and placed on ice. Then, the pod samples were blanched in a 100°C water bath (Mozzoni et al., 2009) and stored in a refrigerator at 1.6°C to maintain freshness until color determination (Tsay and Sheu, 1991).

### Traits Assessed

A description of the traits assessed is as follows:

*PlantingDate_DOY:* Day of year when planting occurred.
*Ve (Day of emergence):* Calendar date when the cotyledons have pulled through the soil surface.
*VeDate_DOY:* Day of year when Ve occurred.
*R1 (Day of first flower):* Calendar date when the first flowers emerged in at least one plant of the plot.
*R1Date_DOY:* Day of year when R1 occurred.
*#Days Ve-R1:* Number of days elapsed between Ve and R1 for a given plot.
*Harvest date:* Calendar date when plot was harvested.
*HarvestDate_DOY:* Day of year when harvest occurred.
*#Days R1-Harvest:* Number of days elapsed between R1 and harvest for a given plot.
*#Days Ve-Harvest:* Number of days elapsed between Ve and harvest for a given plot.
*GDD Ve-R1 (Growing Degree Days to R1):* The Tu was calculated as described by Miller et al. (2001) with a base temperature suggested by Boote et al. (1998) for the days between Ve and R1, with data generated by weather station nearby the trial location.
*GDD Ve-Harvest:* Calculated as described by Miller et al. (2001) using observed thermal units between Ve and Harvest.
*GDD R1-Harvest:* Calculated as described by Miller et al. (2001) using observed thermal units between R1 and day of Harvest.
*IGC (Intensity of Green Color):* Pod color was measured with a HunterLab ColorFlex (Hunter Associates Laboratory Inc., Reston, VA, United States). The instrument was calibrated with a black glass tile and a white standard tile with values of a^∗^ (−0.93) and b^∗^ (1.02). A green standard tile with values of a^∗^ (−25.30) and b^∗^ (13.71) was used to validate the calibration. IGC was calculated as: $IGC=\frac{-a}{b}$
*Hue:* Describes how close a color is to pure red, yellow, green, or blue (values of 0°, 90°, 180°, or 270°, respectively). Pod hue was calculated, as reported by Rayaprolu et al. (2015), using the equation:
  $Hue=arcTan\left(\frac{a}{b}\right)$
*HPW (Hundred-pod weight):* Weight in grams of 100 pods prior to blanching.
*EHQI:* An index was developed to represent into a single trait the maximization values for IGC, HUE, and HPW. With this index, the greater the value, the greater the quality of the edamame pods. EHQI was calculated as:

$$EHQI=\frac{\left(\frac{HPW}{HPWmax^{†}}\right)}{\left(120-HUE\right)*\left(1-IGC\right)}$$

where HPWmax^†^ was calculated as the maximum hundred pod weight for a given cultivar, planting date, and location and year combination.

This index was built considering two key factors in edamame quality, namely, pod color, and pod size. As reported by Wibowo et al. (2020), #1 grade edamame (standard quality) is determined by the number of pods per 500 g (equivalent to our proposed *HPW* measurement), by the appearance of pods that are not too old and yellow, and by the pod color that must be uniformly green, among other factors. The other parameters in Wibowo et al. (2020) edamame grading system are either under heavy genetic control or under environmental effect but not necessarily affected by the crop developmental stage at harvest time (such as damage by pests and diseases or pod shape). Since seed size, and concomitantly *HPW*, is under genotypic control (Xu et al., 2016), in our index, we used a ratio of *HPW* to the maximum *HPW* observed for a given cultivar across all its harvest dates. Also, the denominator of *EHQI* includes a measurement of *HUE* and *IGC* as a means to counter the effect of pod size in the index; *HUE* and *IGC* are multiplicative and placed in the denominator of the index to highlight the greater importance of green color in overall edamame quality as demonstrated by the inclusion of two elements of color in edamame grading systems (Wibowo et al., 2020). *HUE* and *IGC* are highly correlated traits, and even though an alternative option would have been to build *EHQI* index using only one of the traits and weighed it using a square power, the authors decided to utilize both *IGC* and *HUE* in the original building of *EHQI*.

### Statistical Analysis

Each Year and Location combination was aggregated into an “Environment” variable that was considered a random factor in all ANOVA, except when predicting the least-square means to be used for the stepwise and neural network analysis of weather variables, under which Environment had to be assumed a fixed effect.

#### Objective 1. Impact of Cultivar, Planting Date, and Harvest Date on Edamame Pod Color and Pod Weight

The PROC GLIMMIX procedure of SAS 9.4 software (SAS Institute, Cary, NC, United States) was used to analyze *HPW*, *IGC*, and *Hue*, with a model with the following fixed effects. Planting Date was the whole plot, Cultivar was the split-plot, and Harvest Date nested within Planting Date and Cultivar was the split-split plot. The random effects were Environment, Block nested within Environment, and Planting Date by Block nested within Environment. A beta distribution with logit link was used for *IGC* analysis, whereas a normal distribution with identity link was used for the models of *HPW* and *Hue*.

#### Objective 2. Impact of Planting and Harvest Dates on EHQI of Three Soybean Cultivars

Since each cultivar is expected to have its own *HPWmax* because of the genetic control of seed size (Xu et al., 2016), ANOVA for *EHQI* was conducted by cultivar using a model in PROC GLIMMIX of SAS 9.4 with a beta distribution with logit link. For this analysis, Planting Date was whole plot and Harvest Date nested within Planting Date was split plot. The random effects were Environment, Block nested within Environment, and Planting Date by Block nested within Environment.

In addition, to characterize the change in edamame quality over time, regression analysis was conducted for *EHQI* as response of *PlantingDate_DOY*, *HarvestDate_DOY*, and their squared values. A stepwise regression was conducted independently for each cultivar using PROC REG in SAS 9.4, with significance level of 0.15 to enter or remove variables from the linear model and minimum Akaike information criterion (AIC) selection criteria. Modeled parameter estimates were then used to build response surfaces to predict *EHQI* for all days within the planting and harvesting day-of-year used.

#### Objective 3. Prediction of EHQI Based on Phenological Stages and Thermal Units Using Stepwise Regression and Artificial Neural Network Analysis

A PROC GLIMMIX procedure for *EHQI* by cultivar, and with Environment as fixed factor, was used to derive least-square means of *EHQI* by cultivar, environment, planting, and harvest date combinations. Harvest Date nested within Planting Date was the split plot in the analysis, and Block nested within Environment, and Planting Date by Block nested within Environment were random terms. The model was run assuming a beta distribution with logit link. Subsequently, a stepwise regression model in PROC REG in SAS 9.4 was used to predict *EHQI* by Cultivar, with *PlantingDate_DOY*, *PlantingDate_DOY^2^*, *VeDate_DOY*, *R1Date_DOY*, *HarvestDate_DOY*, *HarvestDate_DOY^2^*, *#Days Ve-R1*, *GDD Ve-R1*, *#Days R1-Harvest*, *GDD R1-Harvest*, *#Days Ve-Harvest*, and *GDD Ve-Harvest* as factors entering and leaving the model. Significance level 0.15 was used to enter or remove variables and estimate the linear model with lowest AIC.

Finally, because of a risk of collinearity and/or non-linear responses to some of the variables entering the stepwise procedure, a neural network analysis was conducted using JMP 15.1, executing a random holdback validation and testing multiple different hidden layer structures of TanH, Linear, or Gaussian activation types and two, three, or 10 first and secondary layers. Absolute penalty was implemented, as it was assumed that a few of the variables contribute more than others to the predictive model. The number of tours was set to 1,000 and random seed to 0.5. Factors included in the analysis were *PlantingDate_DOY*, *VeDate_DOY*, *R1Date_DOY*, *HarvestDate_DOY*, *#Days Ve-R1*, *GDD Ve-R1*, *#Days R1-Harvest*, *GDD R1-Harvest*, *#Days Ve-Harvest*, and *GDD Ve-Harvest*, and the response variable was *EHQI.*

## Results

### Impact of Cultivar, Planting Date, and Harvest Date on Edamame Pod Color and Pod Weight

**FIGURE 1:**
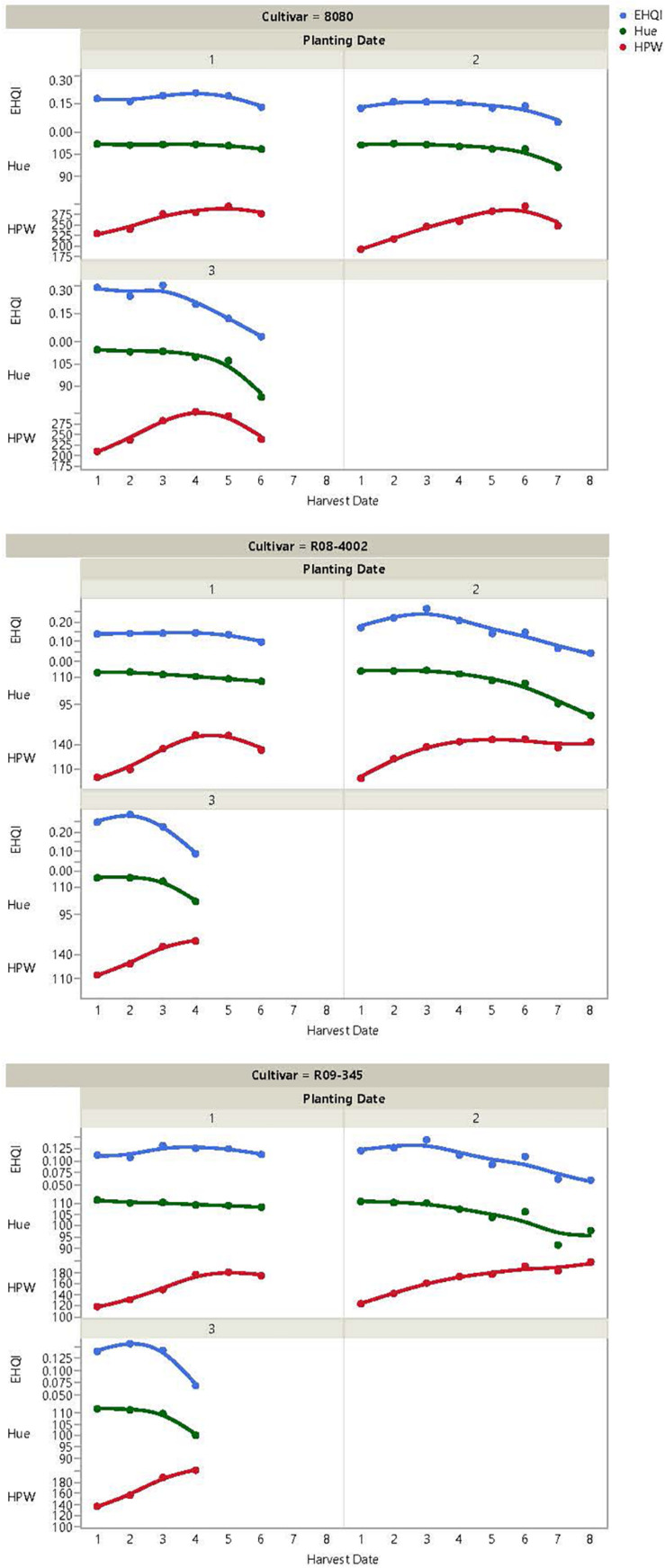
Edamame Harvest Quality Index (EHQI), Hue, and Hundred-Pod Weight (HPW) of three soybean cultivars when planted in three planting dates and subjected to weekly harvest dates, beginning at R5.8 stage and discontinuing at R7 physiological stage.

A split-split-plot analysis for edamame *HPW* indicated a non-significant Planting Date effect (0.2235) or Planting Date by Cultivar interaction (*p* = 0.2040) but significant Cultivar (*p* < 0.0001) and Harvest-Date-by-Planting-Date-by-Cultivar interactions (*p* < 0.0001). Similarly, for *Hue* and *IGC*, the Harvest-Date-by-Planting-Date-by-Cultivar interactions were highly significant (*p* < 0.0001). Those models also indicated significant main effects of planting date (*p* < 0.0001 and *p* = 0.0003 for *Hue* and *IGC*, respectively) and Cultivar (*p* = 0.0036 and *p* < 0.0001 for *Hue* and *IGC*, respectively) and a highly significant interaction with Planting Date-by-Cultivar (*p* < 0.0001 for *Hue* and *p* < 0.0001 for *IGC*). All these results indicated that the responses of *HPW*, *Hue*, and *IGC* must be explored independently by planting date, harvest date, and cultivar combination (Figure 1). Supplementary Tables 1–3 present the least square mean estimates, standard error, and conservative T-grouping for *HPW*, *Hue*, and *IGC*, respectively. In general, it was observed that *HPW* increased over the first four harvest dates. The earliest-maturity cultivar (8080) presented a significant decrease in *HPW* for the second and third planting dates when harvesting extended past Harvest Date 6; such drop in *HPW* was not observed for the later maturity cultivars (Figure 1 and Supplementary Table 1). On the other hand, *Hue* (Figure 1 and Supplementary Table 2) and *IGC* (Supplementary Table 3) showed a decrease with soybean physiological development, and the maximum values were observed at R5.8, corresponding to the first Harvest Date. It is noteworthy that *Hue* and *IGC* are highly correlated traits (*r* = 0.99, *p* < 0.001), and they behaved similarly for all cultivars and planting dates. Future research efforts in edamame may focus on just *IGC* instead of measuring both traits because *IGC* is easier to interpret since the objective is to maximize *IGC* for a dark-green edamame pod product. Finally, Planting Date 2 resulted in the greatest number of weekly harvests possible between R5.8 and R7 for all cultivars, while delayed planting (Planting Date 3) resulted in a rapid reduction in green color (*Hue* and *IGC*) and the crop reached R7 faster for all three cultivars compared to the other planting dates (Figure 1).

### Impact of Planting and Harvest Dates on Edamame Harvest Quality Index of Thee Soybean Cultivars

ANOVA of *EHQI* for the three soybean cultivars showed significant effects of Planting Date (*p* = 0.0003, *p* < 0.0001, and *p* = 0.0356 for 8080, R08-4002, and R09-345, respectively) and highly significant Harvest-Date-by-Planting-Date effects (all three cultivars had *p* < 0.0001). Supplementary Table 4 reports the mean *EHQI* for the interaction of Harvest-Date-by-Planting-Date. Initial inspection of plots of *EHQI* over Harvest-Date-by-Planting-Date (Figure 1 and Supplementary Figures 1–3) showed that the third planting date consistently resulted in the greatest *EHQI* on all three cultivars and that *EHQI* began to decay over harvest date for all planting dates and cultivars. In addition, *EHQI* was highly influenced by pod color, whereby *EHQI* only increased because of an increase in *HPW* if *Hue* (or *IGC*) was at a maximum level. For instance, for Cultivar 8080 and Planting Date 1, it can be observed how *EHQI* increased on the third and fourth Harvest Date as *HPW* increased while *Hue* remained relatively flat (Figure 1). However, when intensity of green pod color decreased, *EHQI* decreased concomitantly, regardless of a potential increase in *HPW*. Such situation can be observed for Cultivar 8080 in Planting Date 3, between Harvest Dates 3 and 4, where there was an increase in *HPW*, a decrease in *Hue*, and a concomitant decrease in *EHQI* (Figure 1).

Stepwise regression analysis retained linear and quadratic terms for *PlantingDate_DOY* and *HarvestDate_DOY* for cultivars 8080 and R09-345, but the linear *PlantingDate_DOY* was non-significant for R08-4002. We observed that regression of *EHQI* using *PlantingDate_DOY*, *HarvestDate_DOY*, *PlantingDate_DOY^2^*, and *HarvestDate_DOY^2^* fitted the data well, with adjusted *R*^2^ values ranging from 34.7 to 50.7% and very low root mean square error (RMSE) (ranging from 0.028 to 0.059), indicating low standard deviations for the unexplained variance in the models (Table 1). Regression parameter estimates showed a quadratic increase of edamame quality with delayed planting; on the contrary, we observed a quadratic decrease of quality with delayed harvesting for all cultivars (Table 1).

**TABLE 1:** Stepwise regression coefficients of cultivar response to Edamame Harvest Quality Index (*EHQI*) based on planting date, harvest date, and their squared values for three edamame cultivars subjected to three planting dates and up to eight harvest dates between stages R5.8 and R7 planted in two Arkansas locations over 2 years.

Cultivar	Intercept	*PlantingDate_DOY*	*PlantingDate_DOY^2^*	*HarvestDate_DOY*	*HarvestDate_DOY^2^*	_RMSE_	Adjusted_R^2^_
8080	−5.21578	−0.02442	0.00008	0.06083	−0.00013	0.04861	0.50740
R08-4002	−22.67342	–	0.00001	0.17232	−0.00033	0.05957	0.42270
R09-345	−9.80640	−0.00970	0.00003	0.08111	−0.00015	0.02823	0.34650

**FIGURE 2:**
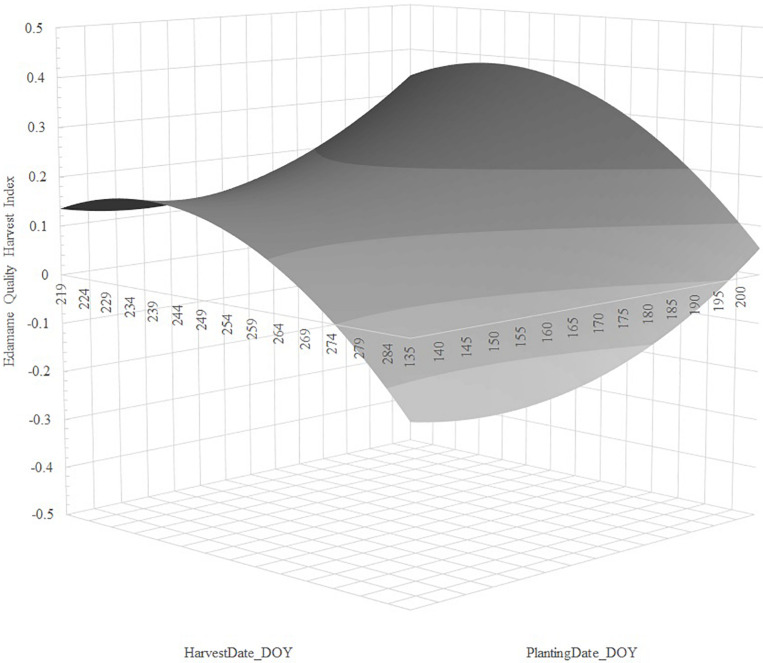
Predicted response of Edamame Harvest Quality Index (*EHQI*) for cultivar 8080, as a function of the regression of *EHQI* on *PlantingDate_DOY*, *PlantingDate_DOY^2^*, *HarvestDate_DOY*, and *HarvestDate_DOY^2^*. Stepwise regression calculated from the dataset of edamame cultivar 8080 planted on two Arkansas locations over 2 years in three planting dates and subjected to up to eight harvests dates, approximately 5 days apart, between R5.8 and R7.

**FIGURE 3:**
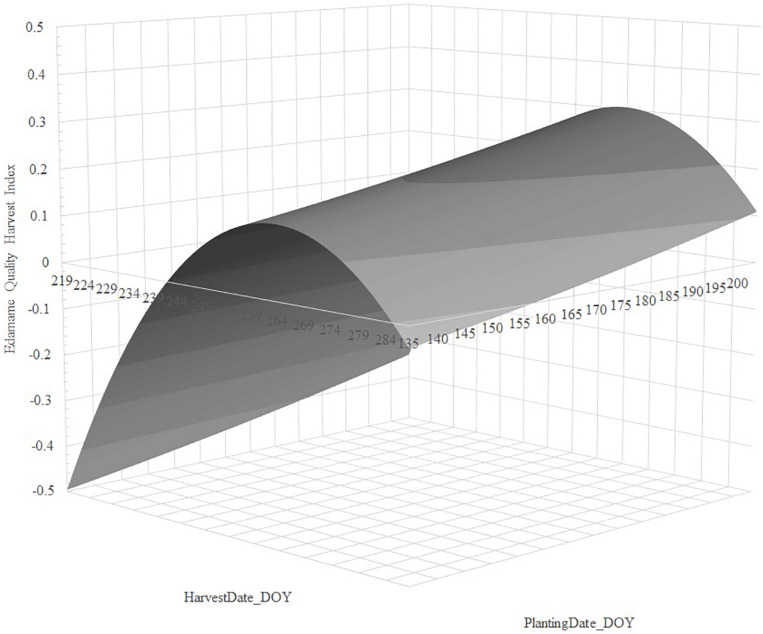
Predicted response of Edamame Harvest Quality Index (*EHQI*) for cultivar R08-4002 as a function of the regression of *EHQI* on *PlantingDate_DOY^2^*, *HarvestDate_DOY*, and *HarvestDate_DOY^2^*. Stepwise regression calculated from the dataset of edamame cultivar R08-4002 planted on two Arkansas locations over 2 years in three planting dates and subjected to up to eight harvests dates, approximately 5 days apart, between R5.8 and R7. *PlantingDate_DOY* was not significant in the stepwise analysis and was not used to build this response surface.

**FIGURE 4:**
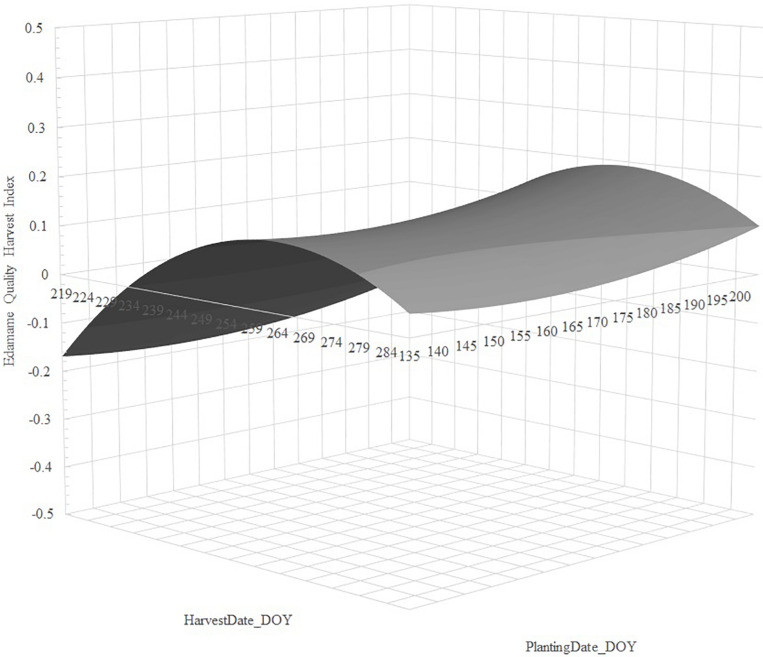
Predicted response of Edamame Harvest Quality Index (*EHQI*) for cultivar R09-345 as a function of the regression of *EHQI* on *PlantingDate_DOY*, *PlantingDate_DOY^2^*, *HarvestDate_DOY*, and *HarvestDate_DOY^2^*. Stepwise regression calculated from dataset of edamame cultivar R09-345 planted on two Arkansas locations over 2 years in three planting dates and subjected to up to eight harvest dates, approximately 5 days apart, between R5.8 and R7.

For cultivar 8080, our data indicated delayed planting increased *EHQI*, and that *EHQI* decreased with delayed harvest; however, near the peak, *EHQI* remained fairly stable (within 0.024 units *EHQI*, or one standard error) for 27 days (Figure 2). For cultivar R08-4002, we also observed that delayed planting resulted in greater *EHQI* and that the quality decreased quadratic with delayed harvest; however, this cultivar did not retain *EHQI* well with delayed harvest, and *EHQI* remained within one standard error (0.026 *EHQI* units) from peak for 18 days (Figure 3). Finally, cultivar R09-345 showed the least total *EHQI* from all cultivars, yet we still observed a quadratic improvement with delayed planting and a quadratic decrease of *EHQI* with delayed harvest (Figure 4). R09-345 had a harvest window around the peak of EHQI that spanned for approximately 20 days, where *EHQI* was within one standard error (or 0.015 *EHQI* units) from peak *EHQI*.

### Prediction of Edamame Harvest Quality Index Based on Phenological Stages and Thermal Units Using Stepwise Regression and Artificial Neural Network Analysis

A stepwise regression and an Artificial Neural Network Analysis were conducted for *EHQI* using variables that included days to various phenological stages and thermal units accumulated to key phenological stages. Stepwise regression models found *#Days R1-Harvest* to be significant across all three cultivars (Table 2), with a negative impact on quality, as the longer period between initiation of flowering and harvest resulted in lower quality. Other significant terms in the model included *#Days Ve-R1* for R09-345 and *#Days Ve-Harvest* for R08-4002. Interestingly enough, the only cultivar that responded to planting date and thermal units was 8080, which is an indeterminate, early cultivar (MG3) and had significant *PlantingDate_DOY*, *VeDate_DOY*, *HarvestDate_DOY^2^*, *GDD Ve-R1*, and *GDD R1-Harvest* (Table 2).

**TABLE 2:** Stepwise regression coefficients of cultivar response to Edamame Harvest Quality Index (*EHQI*) based on phenological and thermal functions for three edamame cultivars subjected to three planting dates and up to eight harvest dates between stages R5.8 and R7 planted in two Arkansas locations over 2 years.

Cultivar	Intercept	*Planting Date_ DOY*	*Ve Date_ DOY*	*Harvest Date_ DOY^2^*	*#Days Ve-R1*	*GDD Ve-R1*	*#Days R1-Harvest*	*GDD R1-Harvest*	*#Days Ve-Harvest*	_ RMSE_	Adjusted_ *R*^2^_
8080	−0.51394	−0.01766	0.02647	−0.00001		−0.00078	−0.02018	0.00131		0.4454	0.5864
R08-4002	0.36379						−0.00765		0.00247	0.0611	0.3933
R09-345	0.11650				0.00205		−0.00171			0.0305	0.2390

A Neural Network model with NTanH parameter of 10 nodes resulted in the lowest RMSE for all three cultivars as compared to models with multiple combinations of TanH, Linear, or Gaussian activation types and two, three, or 10 first and secondary layers (data not shown). For cultivar 8080, we observed that *#Days R1-Harvest* was the most important variable in predicting *EHQI*, with a total effect of 0.503 that was three times larger than the next variable in total effect. Also, for cultivar 8080, the variables with main effect greater than 0.100 included *#Days Ve-Harvest*, *#Days R1-Harvest*, *GDD Ve-Harvest*, *HarvestDate_DOY*, and *GDD Ve-R1* (Table 3). For soybean cultivar R08-4002, we observed that #Days R1-Harvest also had the largest contribution to the predictive model for *EHQI* (total effect 0.314). Additionally, for R08-4002, we observed that the variables with main effects greater than 0.100 included *#Days R1-Harvest*, *GDD R1-Harvest*, *R1Date_DOY*, *VeDate_DOY*, and *PlantingDate_DOY* (Table 4). Lastly, for the prediction of *EHQI* of R09-345, we observed that *#Days Ve-R1* had the largest total effect (0.201) and that the following variables each had main effects greater than 0.100: *HarvestDate_DOY*, *#Days Ve-Harvest*, *#Days R1-Harvest*, *GDD R1-Harvest*, and *R1Date_DOY* (Table 5).

**TABLE 3:** Neural Network Analysis model with NTanH(10) summary of training and validation model and variable importance assuming dependent resampled inputs for the prediction of response variable Edamame Harvest Quality Index (*EHQI*) based on phenological (Ve, emergence; R1, first flower) day-of-year (DOY), and thermal functions (GDD, growing degree days) for soybean cultivar 8080.

Model summary	Training	Validation
*R*^2^	0.842	0.735
RMSE	0.029	0.032

**Variable importance**	**Main effect**	**Total effect**

*#Days R1-Harvest*	0.119	0.503
*#Days Ve-Harvest*	0.162	0.162
*GDD Ve-Harvest*	0.106	0.114
*HarvestDate_DOY*	0.109	0.109
*GDD Ve-R1*	0.102	0.102
*GDD R1-Harvest*	0.084	0.084
*PlantingDate_DOY*	0.082	0.082
*VeDate_DOY*	0.082	0.082
*R1Date_DOY*	0.081	0.081
*#Days Ve-R1*	0.073	0.073

**TABLE 4:** Neural Network Analysis model with NTanH(10) summary of training and validation model and variable importance assuming dependent resampled inputs for the prediction of response variable Edamame Harvest Quality Index (*EHQI*) based on phenological (Ve, emergence; R1, first flower) day-of-year (DOY), and thermal functions (GDD, growing degree days) for soybean cultivar R08-4002.

Model summary	Training	Validation
*R*^2^	0.703	0.591
RMSE	0.046	0.039

**Variable importance**	**Main effect**	**Total effect**

*#Days R1-Harvest*	0.171	0.314
*GDD R1-Harvest*	0.122	0.122
*R1Date_DOY*	0.108	0.108
*VeDate_DOY*	0.104	0.104
*PlantingDate_DOY*	0.103	0.103
*GDD Ve-Harvest*	0.098	0.098
*GDD Ve-R1*	0.080	0.080
*HarvestDate_DOY*	0.073	0.073
*#Days Ve-Harvest*	0.074	0.074
*#Days Ve-R1*	0.066	0.066

**TABLE 5:** Neural Network Analysis model with NTanH(10) summary of training and validation model and variable importance assuming dependent resampled inputs for the prediction of response variable Edamame Harvest Quality Index (*EHQI*) based on phenological (Ve, emergence; R1, first flower) day-of-year (DOY), and thermal functions (GDD, growing degree days) for soybean cultivar R09-345.

Model summary	Training	Validation
*R*^2^	0.668	0.783
RMSE	0.017	0.020

**Variable importance**	**Main effect**	**Total effect**

*#Days Ve-R1*	0.069	0.201
*HarvestDate_DOY*	0.155	0.155
*#Days Ve-Harvest*	0.135	0.135
*#Days R1-Harvest*	0.132	0.132
*GDD Ve-Harvest*	0.082	0.109
*GDD R1-Harvest*	0.103	0.103
*R1Date_DOY*	0.100	0.100
*PlantingDate_DOY*	0.082	0.082
*VeDate_DOY*	0.078	0.078
*GDD Ve-R1*	0.064	0.064

## Discussion

High edamame quality is characterized by large pod size and intense and uniform green pod color (Wibowo et al., 2020). Panthee et al. (2004) suggested that seed size has high heritability, indicating that the trait should be controlled more by genetic than environmental variances. A study by Beatty et al. (1982) found that seed weight was not significantly different from April 15 to May 15 planting date but dropped significantly each month from a May 15 to July 15 planting date. Similarly, in our study, we found that *HPW* depended on the interaction between planting date and harvest date and that *HPW* increased during the first four Harvest Dates for all cultivars and planting dates.

The second component of edamame quality is intensity of pod green color. Of the cultivars chosen for our study, R09-345 has green seeds at R6 but develops black seed coat color at maturity. Such cultivar showed significantly lower pod *Hue* and *IGC* and low overall *EHQI* than the other two cultivars (Figure 4 and Supplementary Tables 2, 3), suggesting that cultivars whose seed turn black or brown at maturity will not have the same pod color at R6 compared to cultivars that either stay green or turn yellow at the R8 reproductive stage. Thus, cultivars with dark seed coat at maturity may not be appropriate for fresh/frozen market edamame production.

We observed that, in general, delayed planting maximized *EHQI*. Mean separations for *EHQI* analyzed as a split-plot design (Supplementary Table 4) showed that the first harvest dates were usually not statistically different from each other, but that as harvest was delayed, *EHQI* dropped. We also observed a quadratic decrease of *EHQI* with delayed harvest in all cultivars from our regression analysis. The harvest window seemed planting date and cultivar dependent. The window for maximum *EHQI* was shorter in late plantings than in earlier planting dates (Supplementary Figures 1–3). Near the cultivar optimum, harvest window for *EHQI* ranged from 18 to 27 days. The low end of the spectrum agrees with the 18-day window reported by Nolen et al. (2016), but cultivar 8080 showed a much larger window where quality did not drop. Therefore, edamame companies aiming for high quality must procure late planting and ensure logistics are in place for earlier and timely harvests 10–15 days after R5.8 stage is observed. This must be achieved by carefully planning the logistics of field equipment availability, field access under unfavorable weather/road conditions, and processing house turnaround times. On the contrary, early plantings maintain quality for longer periods of time, albeit not maximizing *EHQI*; therefore, edamame-growing companies could target earlier plantings to marginal grounds, farms with difficult access, or periods where the processing plant is at its peak, all while managing the maturity of the cultivar to spread out flowering (and harvest) timelines.

The early maturing indeterminate cultivar 8080 showed a significant response to thermal units (*GDD Ve-R1* and *GDD R1-Harvest*) in stepwise regression analysis, while the late-maturity cultivars did not. This agrees with expected soybean response where the temperature is considered a modifier to the effect of photoperiod whereby short days enhance reproductive development rate (Setiyono et al., 2007) and with the expected insensitivity of earlier maturity to photoperiod (Salmeron et al., 2014), thus enhancing the opportunity for temperature responses.

**FIGURE 5:**
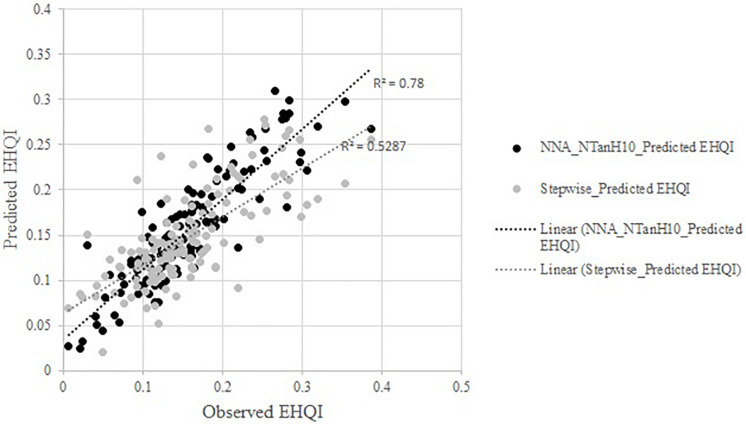
Plot of predicted Edamame Harvest Quality Index (*EHQI*) by Neural Network Analysis and stepwise analysis as a function of the observed *EHQI*. Neural network model with NTanH(10) assuming dependent resampled inputs. Both models utilized phenological and thermal functions for the prediction of *EHQI*.

Both stepwise regression and Neural Network Analysis identified *#Days R1-Harvest* as a key variable affecting *EHQI* for all three soybean cultivars. Additionally, when looking at the prediction values for *EHQI* based on Neural Network and stepwise analyses (Figure 5), we observed that formulas from stepwise analysis tended to overpredict lower and underpredict higher *EHQI* values, whereas Neural Network prediction was more consistent over the range of data. However, the simplicity of stepwise predictions involving a simple model with few parameters for cultivars of maturities adapted to Arkansas could make field assessments easier. Future research is needed to test these prediction models on other field-grown edamame cultivars to explore their applicability in forecasting harvest decisions.

Finally, and even though our research did not study pod or seed yield, it is a very important criterion for edamame farmers as they need to balance yield and quality of their end product. Therefore, agronomic practices must be used to balance increased seed and pod yield resulting from earlier planting dates (De Bruin and Pedersen, 2008; Mourtzinis et al., 2017) and enhanced seed and pod quality observed from later planting dates.

## Data Availability Statement

The raw data supporting the conclusions of this article will be made available by the authors, without undue reservation.

## Author Contributions

DM was responsible for the investigation, methodology, and writing of the original draft. LM was responsible for supervision, data curation, data analysis, and editing and reviewing the final document. MS, MO, LF-P, AA, and CW were responsible for the investigation and editing and reviewing the final document. PC acquired funding and conceptualized the project and edited and reviewed the final document. All authors contributed to the article and approved the submitted version.

## Conflict of Interest

The authors declare that the research was conducted in the absence of any commercial or financial relationships that could be construed as a potential conflict of interest.

## Supplementary Material

Supplementary Figure 1 Edamame Harvest Quality Index (*EHQI*) as function of harvest date treatment by planting date treatment for soybean cultivar 8080. Error bars represent standard errors of least-square means for *EHQI*.

Supplementary Figure 2 Edamame Harvest Quality Index (*EHQI*) as function of harvest date by planting date for soybean breeding cultivar R08-4002. Error bars represent standard errors of least-square means for *EHQI*.

Supplementary Figure 3 Edamame Harvest Quality Index (*EHQI*) as function of harvest date by planting date for soybean breeding cultivar R09-345. Error bars represent standard errors of least-square means for *EHQI*.

Supplementary Table 1 Least-square means, Standard Error, and Conservative T-grouping of Hundred Pod Weight (*HPW*) per cultivar, planting and harvest date combination analyzed on a split-split block design with block and environment as random factors. Levels not connected by same letter are significantly different at α = 0.05.

Supplementary Table 2 Least-square means, Standard Error, and Conservative T-grouping of *Hue* per cultivar, planting and harvest date combination analyzed on a split-split block design with block and environment as random factors. Levels not connected by same letter are significantly different at α = 0.05.

Supplementary Table 3 Least-square means, Standard Error, and Conservative T-grouping of Intensity of Green Color (*IGC*) per cultivar, planting and harvest date combination analyzed on a split-split block design with block and environment as random factors. Levels not connected by same letter are significantly different at α = 0.05.

Supplementary Table 4 Least-square mean, Standard Error, and Conservative T-grouping of Edamame Harvest Quality Index (*EHQI*) by cultivar, per planting and harvest date combination, analyzed as a split block design with block and environment as random factors. Levels not connected by same letter are significantly different at α = 0.05. **(A)** R08-4002, **(B)** 8080, and **(C)** R09-345.
