# Supplementary material for: Effect of Planting Date and Cultivar Maturity in Edamame Quality and Harvest Window
Source: Front Plant Sci. 2021 Jan 18;11:585856. doi: 10.3389/fpls.2020.585856 (PMC7847894; doi:10.3389/fpls.2020.585856)

**Effect of Planting Date and Cultivar Maturity in Edamame Quality and Harvest Window**

David Moseley^1^, Marcos Paulo da Silva^2^, Leandro Mozzoni^2 *^, Moldir Orazaly^2^, Liliana Florez-Palacios^2^, Andrea Acuña^2^, Chengjun Wu^2^, and Pengyin Chen^3^

^1^ LSU AgCenter, 8105 Tom Bowman Drive, Alexandria, LA 71302, USA

^2^ Department of Crop, Soil, and Environmental Sciences, University of Arkansas, Fayetteville, AR 72701, USA

^3^ Division of Plant Sciences, 1-31 Ag Building, University of Missouri, Columbia, MO 65201, USA

###### ^*^Correspondence:

Dr. Leandro Mozzoni

[lmozzon@uark.edu](mailto:lmozzon@uark.edu)

**Supplementary Table S1.** Least-square means, Standard Error, and Conservative T-grouping of Hundred Pod Weight (*HPW*) per cultivar, planting and harvest date combination analyzed on a split-split block design with block and environment as random factors. Levels not connected by same letter are significantly different at α= 0.05.

| **Planting Date** | **Cultivar** | **Harvest Date** | **Mean** | **Standard Error** |  |  |  |  |  |  |  |  |  |  |  |  |  |  |  |  |  |  |  |  |  |  |  |  |
| --- | --- | --- | --- | --- | --- | --- | --- | --- | --- | --- | --- | --- | --- | --- | --- | --- | --- | --- | --- | --- | --- | --- | --- | --- | --- | --- | --- | --- |
| 3 | 8080 | 4 | 306.26 | 8.85 | A |  |  |  |  |  |  |  |  |  |  |  |  |  |  |  |  |  |  |  |  |  |  |  |
| 3 | 8080 | 5 | 296.26 | 8.85 | A | B |  |  |  |  |  |  |  |  |  |  |  |  |  |  |  |  |  |  |  |  |  |  |
| 2 | 8080 | 6 | 293.51 | 8.81 | A | B |  |  |  |  |  |  |  |  |  |  |  |  |  |  |  |  |  |  |  |  |  |  |
| 1 | 8080 | 5 | 293.14 | 10.83 | A | B |  |  |  |  |  |  |  |  |  |  |  |  |  |  |  |  |  |  |  |  |  |  |
| 3 | 8080 | 3 | 284.82 | 8.85 |  | B | C |  |  |  |  |  |  |  |  |  |  |  |  |  |  |  |  |  |  |  |  |  |
| 2 | 8080 | 5 | 282.14 | 8.81 |  | B | C | D |  |  |  |  |  |  |  |  |  |  |  |  |  |  |  |  |  |  |  |  |
| 1 | 8080 | 4 | 278.82 | 10.83 |  | B | C | D |  |  |  |  |  |  |  |  |  |  |  |  |  |  |  |  |  |  |  |  |
| 1 | 8080 | 6 | 276.12 | 10.83 |  | B | C | D | E |  |  |  |  |  |  |  |  |  |  |  |  |  |  |  |  |  |  |  |
| 1 | 8080 | 3 | 275.39 | 10.83 |  | B | C | D | E |  |  |  |  |  |  |  |  |  |  |  |  |  |  |  |  |  |  |  |
| 3 | 8080 | 7 | 259.53 | 13.32 |  |  | C | D | E | F |  |  |  |  |  |  |  |  |  |  |  |  |  |  |  |  |  |  |
| 2 | 8080 | 4 | 258.29 | 8.81 |  |  |  |  | E | F |  |  |  |  |  |  |  |  |  |  |  |  |  |  |  |  |  |  |
| 2 | 8080 | 7 | 247.57 | 10.12 |  |  |  |  | E | F |  |  |  |  |  |  |  |  |  |  |  |  |  |  |  |  |  |  |
| 2 | 8080 | 3 | 245.84 | 8.81 |  |  |  |  |  | F |  |  |  |  |  |  |  |  |  |  |  |  |  |  |  |  |  |  |
| 3 | 8080 | 6 | 241.49 | 10.17 |  |  |  |  |  | F | G |  |  |  |  |  |  |  |  |  |  |  |  |  |  |  |  |  |
| 1 | 8080 | 2 | 239.95 | 10.83 |  |  |  |  |  | F | G |  |  |  |  |  |  |  |  |  |  |  |  |  |  |  |  |  |
| 3 | 8080 | 2 | 239.66 | 8.85 |  |  |  |  |  | F | G |  |  |  |  |  |  |  |  |  |  |  |  |  |  |  |  |  |
| 1 | 8080 | 1 | 229.35 | 10.83 |  |  |  |  |  | F | G | H |  |  |  |  |  |  |  |  |  |  |  |  |  |  |  |  |
| 2 | 8080 | 2 | 216.80 | 8.81 |  |  |  |  |  |  | G | H | I |  |  |  |  |  |  |  |  |  |  |  |  |  |  |  |
| 3 | 8080 | 1 | 212.82 | 8.85 |  |  |  |  |  |  |  | H | I | J |  |  |  |  |  |  |  |  |  |  |  |  |  |  |
| 3 | R09-345 | 4 | 204.04 | 8.85 |  |  |  |  |  |  |  | H | I | J | K |  |  |  |  |  |  |  |  |  |  |  |  |  |
| 2 | R09-345 | 8 | 198.98 | 13.02 |  |  |  |  |  |  |  | H | I | J | K |  |  |  |  |  |  |  |  |  |  |  |  |  |
| 2 | 8080 | 1 | 192.30 | 8.81 |  |  |  |  |  |  |  |  | I | J | K | L |  |  |  |  |  |  |  |  |  |  |  |  |
| 2 | R09-345 | 6 | 191.98 | 9.82 |  |  |  |  |  |  |  |  | I | J | K | L |  |  |  |  |  |  |  |  |  |  |  |  |
| 3 | R09-345 | 3 | 190.74 | 8.85 |  |  |  |  |  |  |  |  |  | J | K | L |  |  |  |  |  |  |  |  |  |  |  |  |
| 2 | R09-345 | 7 | 182.98 | 9.82 |  |  |  |  |  |  |  |  |  |  | K | L | M |  |  |  |  |  |  |  |  |  |  |  |
| 1 | R09-345 | 5 | 180.75 | 10.83 |  |  |  |  |  |  |  |  |  |  | K | L | M | N |  |  |  |  |  |  |  |  |  |  |
| 2 | R09-345 | 5 | 176.93 | 8.44 |  |  |  |  |  |  |  |  |  |  | K | L | M | N | O |  |  |  |  |  |  |  |  |  |
| 1 | R09-345 | 4 | 176.40 | 10.83 |  |  |  |  |  |  |  |  |  |  | K | L | M | N | O | P |  |  |  |  |  |  |  |  |
| 1 | R09-345 | 6 | 174.56 | 13.87 |  |  |  |  |  |  |  |  |  |  | K | L | M | N | O | P |  |  |  |  |  |  |  |  |
| 2 | R09-345 | 4 | 172.79 | 7.63 |  |  |  |  |  |  |  |  |  |  |  | L | M | N | O | P |  |  |  |  |  |  |  |  |
| 2 | R09-345 | 3 | 161.23 | 7.63 |  |  |  |  |  |  |  |  |  |  |  |  | M | N | O | P | Q |  |  |  |  |  |  |  |
| 3 | R09-345 | 2 | 158.93 | 8.85 |  |  |  |  |  |  |  |  |  |  |  |  | M | N | O | P | Q |  |  |  |  |  |  |  |
| 3 | 4002 | 4 | 158.52 | 8.85 |  |  |  |  |  |  |  |  |  |  |  |  | M | N | O | P | Q |  |  |  |  |  |  |  |
| 1 | 4002 | 4 | 152.24 | 10.83 |  |  |  |  |  |  |  |  |  |  |  |  |  | N | O | P | Q | R |  |  |  |  |  |  |
| 1 | 4002 | 5 | 151.77 | 10.83 |  |  |  |  |  |  |  |  |  |  |  |  |  |  | O | P | Q | R |  |  |  |  |  |  |
| 3 | 4002 | 3 | 151.68 | 8.85 |  |  |  |  |  |  |  |  |  |  |  |  |  |  |  | P | Q | R |  |  |  |  |  |  |
| 1 | R09-345 | 3 | 149.30 | 10.83 |  |  |  |  |  |  |  |  |  |  |  |  |  |  |  | P | Q | R |  |  |  |  |  |  |
| 2 | 4002 | 6 | 147.26 | 9.82 |  |  |  |  |  |  |  |  |  |  |  |  |  |  |  | P | Q | R | S |  |  |  |  |  |
| 2 | 4002 | 5 | 146.44 | 8.44 |  |  |  |  |  |  |  |  |  |  |  |  |  |  |  | P | Q | R | S |  |  |  |  |  |
| 2 | 4002 | 4 | 143.91 | 7.63 |  |  |  |  |  |  |  |  |  |  |  |  |  |  |  | P | Q | R | S | T |  |  |  |  |
| 2 | 4002 | 8 | 143.60 | 13.02 |  |  |  |  |  |  |  |  |  |  |  |  |  |  |  | P | Q | R | S | T | U |  |  |  |
| 2 | R09-345 | 2 | 142.25 | 7.63 |  |  |  |  |  |  |  |  |  |  |  |  |  |  |  |  | Q | R | S | T | U |  |  |  |
| 3 | R09-345 | 1 | 138.83 | 8.85 |  |  |  |  |  |  |  |  |  |  |  |  |  |  |  |  | Q | R | S | T | U |  |  |  |
| 2 | 4002 | 3 | 137.09 | 7.63 |  |  |  |  |  |  |  |  |  |  |  |  |  |  |  |  | Q | R | S | T | U |  |  |  |
| 2 | 4002 | 7 | 136.42 | 9.82 |  |  |  |  |  |  |  |  |  |  |  |  |  |  |  |  | Q | R | S | T | U | V |  |  |
| 1 | 4002 | 3 | 134.79 | 10.83 |  |  |  |  |  |  |  |  |  |  |  |  |  |  |  |  | Q | R | S | T | U | V |  |  |
| 1 | 4002 | 6 | 133.22 | 13.87 |  |  |  |  |  |  |  |  |  |  |  |  |  |  |  |  | Q | R | S | T | U | V |  |  |
| 1 | R09-345 | 2 | 131.17 | 10.83 |  |  |  |  |  |  |  |  |  |  |  |  |  |  |  |  |  | R | S | T | U | V |  |  |
| 3 | 4002 | 2 | 129.63 | 8.85 |  |  |  |  |  |  |  |  |  |  |  |  |  |  |  |  |  | R | S | T | U | V |  |  |
| 2 | R09-345 | 1 | 123.87 | 7.63 |  |  |  |  |  |  |  |  |  |  |  |  |  |  |  |  |  |  | S | T | U | V |  |  |
| 2 | 4002 | 2 | 122.05 | 7.63 |  |  |  |  |  |  |  |  |  |  |  |  |  |  |  |  |  |  |  | T | U | V | W |  |
| 1 | R09-345 | 1 | 118.85 | 10.83 |  |  |  |  |  |  |  |  |  |  |  |  |  |  |  |  |  |  |  | T | U | V | W | X |
| 3 | 4002 | 1 | 115.22 | 8.85 |  |  |  |  |  |  |  |  |  |  |  |  |  |  |  |  |  |  |  |  | U | V | W | X |
| 1 | 4002 | 2 | 108.55 | 10.83 |  |  |  |  |  |  |  |  |  |  |  |  |  |  |  |  |  |  |  |  |  | V | W | X |
| 1 | 4002 | 1 | 98.50 | 10.83 |  |  |  |  |  |  |  |  |  |  |  |  |  |  |  |  |  |  |  |  |  |  | W | X |
| 2 | 4002 | 1 | 97.23 | 7.63 |  |  |  |  |  |  |  |  |  |  |  |  |  |  |  |  |  |  |  |  |  |  |  | X |

**Supplemental Table S2.** Least-square means, Standard Error, and Conservative T-grouping of *Hue* per cultivar, planting and harvest date combination analyzed on a split-split block design with block and environment as random factors. Levels not connected by same letter are significantly different at α= 0.05.

| **Planting Date** | **Cultivar** | **Harvest Date** | **Mean** | **Standard Error** |  |  |  |  |  |  |  |  |  |  |  |  |  |
| --- | --- | --- | --- | --- | --- | --- | --- | --- | --- | --- | --- | --- | --- | --- | --- | --- | --- |
| 3 | 8080 | 1 | 115.92 | 1.72 | A |  |  |  |  |  |  |  |  |  |  |  |  |
| 3 | 4002 | 2 | 115.43 | 2.04 | A | B |  |  |  |  |  |  |  |  |  |  |  |
| 3 | 4002 | 1 | 115.33 | 1.72 | A | B |  |  |  |  |  |  |  |  |  |  |  |
| 3 | 8080 | 3 | 114.77 | 1.72 | A | B |  |  |  |  |  |  |  |  |  |  |  |
| 3 | 8080 | 2 | 114.49 | 1.72 | A | B | C |  |  |  |  |  |  |  |  |  |  |
| 2 | 4002 | 3 | 113.55 | 1.53 | A | B | C | D |  |  |  |  |  |  |  |  |  |
| 3 | 4002 | 3 | 113.51 | 1.72 | A | B | C | D | E |  |  |  |  |  |  |  |  |
| 2 | 4002 | 2 | 113.22 | 1.53 | A | B | C | D | E |  |  |  |  |  |  |  |  |
| 2 | 4002 | 1 | 113.13 | 1.53 | A | B | C | D | E |  |  |  |  |  |  |  |  |
| 2 | 8080 | 2 | 112.74 | 1.71 | A | B | C | D | E |  |  |  |  |  |  |  |  |
| 1 | 4002 | 2 | 112.68 | 2.04 | A | B | C | D | E | F |  |  |  |  |  |  |  |
| 1 | 8080 | 1 | 112.37 | 2.04 | A | B | C | D | E | F |  |  |  |  |  |  |  |
| 1 | 4002 | 1 | 112.33 | 2.04 | A | B | C | D | E | F |  |  |  |  |  |  |  |
| 3 | R09-345 | 1 | 112.18 | 1.72 | A | B | C | D | E | F |  |  |  |  |  |  |  |
| 1 | 8080 | 4 | 112.10 | 2.04 | A | B | C | D | E | F |  |  |  |  |  |  |  |
| 2 | 8080 | 3 | 112.02 | 1.71 | A | B | C | D | E | F |  |  |  |  |  |  |  |
| 1 | 8080 | 3 | 111.98 | 2.04 | A | B | C | D | E | F |  |  |  |  |  |  |  |
| 3 | R09-345 | 2 | 111.69 | 2.04 | A | B | C | D | E | F |  |  |  |  |  |  |  |
| 2 | 4002 | 4 | 111.65 | 1.53 | A | B | C | D | E | F |  |  |  |  |  |  |  |
| 2 | 8080 | 1 | 111.62 | 1.71 | A | B | C | D | E | F |  |  |  |  |  |  |  |
| 1 | 8080 | 2 | 111.59 | 2.04 | A | B | C | D | E | F |  |  |  |  |  |  |  |
| 1 | 4002 | 3 | 111.32 | 2.04 | A | B | C | D | E | F |  |  |  |  |  |  |  |
| 1 | R09-345 | 1 | 111.30 | 2.04 | A | B | C | D | E | F |  |  |  |  |  |  |  |
| 1 | 8080 | 5 | 111.22 | 2.04 | A | B | C | D | E | F |  |  |  |  |  |  |  |
| 3 | 8080 | 4 | 110.77 | 1.72 |  | B | C | D | E | F |  |  |  |  |  |  |  |
| 2 | R09-345 | 1 | 110.64 | 1.53 |  | B | C | D | E | F |  |  |  |  |  |  |  |
| 2 | 8080 | 4 | 110.55 | 1.71 |  | B | C | D | E | F |  |  |  |  |  |  |  |
| 1 | 4002 | 4 | 110.29 | 2.04 |  | B | C | D | E | F |  |  |  |  |  |  |  |
| 1 | R09-345 | 3 | 110.25 | 2.04 |  | B | C | D | E | F |  |  |  |  |  |  |  |
| 3 | R09-345 | 3 | 110.12 | 1.72 |  |  | C | D | E | F |  |  |  |  |  |  |  |
| 2 | R09-345 | 2 | 110.11 | 1.53 |  |  | C | D | E | F |  |  |  |  |  |  |  |
| 2 | R09-345 | 3 | 110.03 | 1.53 |  |  | C | D | E | F |  |  |  |  |  |  |  |
| 1 | R09-345 | 2 | 109.99 | 2.04 |  |  | C | D | E | F |  |  |  |  |  |  |  |
| 1 | R09-345 | 4 | 109.15 | 2.04 |  |  |  | D | E | F |  |  |  |  |  |  |  |
| 1 | 4002 | 5 | 108.97 | 2.04 |  |  |  | D | E | F |  |  |  |  |  |  |  |
| 2 | 8080 | 5 | 108.88 | 1.71 |  |  |  | D | E | F |  |  |  |  |  |  |  |
| 2 | 8080 | 6 | 108.84 | 2.04 |  |  |  | D | E | F |  |  |  |  |  |  |  |
| 1 | R09-345 | 5 | 108.84 | 1.71 |  |  |  | D | E | F |  |  |  |  |  |  |  |
| 1 | 8080 | 6 | 108.74 | 2.04 |  |  |  | D | E | F |  |  |  |  |  |  |  |
| 3 | 8080 | 5 | 108.29 | 1.72 |  |  |  | D | E | F |  |  |  |  |  |  |  |
| 1 | R09-345 | 6 | 108.16 | 2.78 |  |  |  | D | E | F | G |  |  |  |  |  |  |
| 2 | 4002 | 5 | 108.07 | 1.71 |  |  |  |  | E | F | G |  |  |  |  |  |  |
| 1 | 4002 | 6 | 107.61 | 2.78 |  |  |  |  | E | F | G |  |  |  |  |  |  |
| 2 | R09-345 | 4 | 107.24 | 1.53 |  |  |  |  |  | F | G |  |  |  |  |  |  |
| 2 | 4002 | 6 | 106.60 | 2.81 |  |  |  |  |  | F | G | H |  |  |  |  |  |
| 2 | R09-345 | 6 | 106.14 | 2.81 |  |  |  |  |  | F | G | H |  |  |  |  |  |
| 2 | R09-345 | 5 | 103.69 | 1.71 |  |  |  |  |  |  | G | H | I |  |  |  |  |
| 3 | 4002 | 4 | 102.67 | 1.72 |  |  |  |  |  |  | G | H | I |  |  |  |  |
| 3 | R09-345 | 4 | 100.64 | 1.72 |  |  |  |  |  |  |  | H | I | J |  |  |  |
| 2 | R09-345 | 8 | 97.85 | 2.81 |  |  |  |  |  |  |  |  | I | J | K |  |  |
| 2 | 8080 | 7 | 96.20 | 2.04 |  |  |  |  |  |  |  |  |  | J | K |  |  |
| 2 | 4002 | 7 | 95.76 | 2.04 |  |  |  |  |  |  |  |  |  |  | K | L |  |
| 2 | R09-345 | 7 | 91.57 | 2.21 |  |  |  |  |  |  |  |  |  |  | K | L |  |
| 2 | 4002 | 8 | 89.46 | 2.81 |  |  |  |  |  |  |  |  |  |  |  | L | M |
| 3 | 8080 | 6 | 82.93 | 2.04 |  |  |  |  |  |  |  |  |  |  |  |  | M |

**Supplemental Table S3.** Least-square means, Standard Error, and Conservative T-grouping of Intensity of Green Color (*IGC*) per cultivar, planting and harvest date combination analyzed on a split-split block design with block and environment as random factors. Levels not connected by same letter are significantly different at α= 0.05.

| **Planting Date** | **Cultivar** | **Harvest Date** | **Mean** | **Standard Error** |  |  |  |  |  |  |  |  |  |  |  |  |  |  |  |  |  |  |  |
| --- | --- | --- | --- | --- | --- | --- | --- | --- | --- | --- | --- | --- | --- | --- | --- | --- | --- | --- | --- | --- | --- | --- | --- |
| 3 | 8080 | 1 | 0.481 | 0.022 | A |  |  |  |  |  |  |  |  |  |  |  |  |  |  |  |  |  |  |
| 3 | 4002 | 1 | 0.469 | 0.022 | A | B |  |  |  |  |  |  |  |  |  |  |  |  |  |  |  |  |  |
| 3 | 4002 | 2 | 0.468 | 0.025 | A | B | C |  |  |  |  |  |  |  |  |  |  |  |  |  |  |  |  |
| 3 | 8080 | 3 | 0.457 | 0.022 | A | B | C | D |  |  |  |  |  |  |  |  |  |  |  |  |  |  |  |
| 3 | 8080 | 2 | 0.451 | 0.022 | A | B | C | D | E |  |  |  |  |  |  |  |  |  |  |  |  |  |  |
| 2 | 4002 | 3 | 0.436 | 0.019 | A | B | C | D | E | F |  |  |  |  |  |  |  |  |  |  |  |  |  |
| 3 | 4002 | 3 | 0.431 | 0.022 |  | B | C | D | E | F |  |  |  |  |  |  |  |  |  |  |  |  |  |
| 2 | 4002 | 2 | 0.429 | 0.019 |  | B | C | D | E | F |  |  |  |  |  |  |  |  |  |  |  |  |  |
| 2 | 4002 | 1 | 0.427 | 0.019 |  | B | C | D | E | F |  |  |  |  |  |  |  |  |  |  |  |  |  |
| 2 | 8080 | 2 | 0.422 | 0.021 |  | B | C | D | E | F | G |  |  |  |  |  |  |  |  |  |  |  |  |
| 1 | 4002 | 2 | 0.412 | 0.025 |  | B | C | D | E | F | G | H |  |  |  |  |  |  |  |  |  |  |  |
| 2 | 8080 | 3 | 0.408 | 0.021 |  |  | C | D | E | F | G | H |  |  |  |  |  |  |  |  |  |  |  |
| 1 | 4002 | 1 | 0.406 | 0.025 |  |  | C | D | E | F | G | H |  |  |  |  |  |  |  |  |  |  |  |
| 1 | 8080 | 1 | 0.406 | 0.025 |  |  |  | D | E | F | G | H |  |  |  |  |  |  |  |  |  |  |  |
| 3 | R09-345 | 1 | 0.403 | 0.021 |  |  |  | D | E | F | G | H |  |  |  |  |  |  |  |  |  |  |  |
| 1 | 8080 | 4 | 0.400 | 0.025 |  |  |  | D | E | F | G | H | I |  |  |  |  |  |  |  |  |  |  |
| 2 | 8080 | 1 | 0.400 | 0.021 |  |  |  |  | E | F | G | H | I |  |  |  |  |  |  |  |  |  |  |
| 1 | 8080 | 3 | 0.398 | 0.025 |  |  |  |  | E | F | G | H | I | J |  |  |  |  |  |  |  |  |  |
| 2 | 4002 | 4 | 0.397 | 0.019 |  |  |  |  |  | F | G | H | I | J |  |  |  |  |  |  |  |  |  |
| 3 | R09-345 | 2 | 0.392 | 0.025 |  |  |  |  |  | F | G | H | I | J | K |  |  |  |  |  |  |  |  |
| 1 | 8080 | 2 | 0.390 | 0.025 |  |  |  |  |  | F | G | H | I | J | K |  |  |  |  |  |  |  |  |
| 1 | 4002 | 3 | 0.384 | 0.025 |  |  |  |  |  | F | G | H | I | J | K | L |  |  |  |  |  |  |  |
| 1 | R09-345 | 1 | 0.384 | 0.025 |  |  |  |  |  | F | G | H | I | J | K | L |  |  |  |  |  |  |  |
| 1 | 8080 | 5 | 0.382 | 0.025 |  |  |  |  |  | F | G | H | I | J | K | L |  |  |  |  |  |  |  |
| 2 | 8080 | 4 | 0.379 | 0.021 |  |  |  |  |  |  | G | H | I | J | K | L |  |  |  |  |  |  |  |
| 3 | 8080 | 4 | 0.377 | 0.021 |  |  |  |  |  |  | G | H | I | J | K | L |  |  |  |  |  |  |  |
| 2 | R09-345 | 1 | 0.377 | 0.019 |  |  |  |  |  |  |  | H | I | J | K | L |  |  |  |  |  |  |  |
| 2 | R09-345 | 2 | 0.367 | 0.019 |  |  |  |  |  |  |  | H | I | J | K | L | M |  |  |  |  |  |  |
| 2 | R09-345 | 3 | 0.365 | 0.018 |  |  |  |  |  |  |  | H | I | J | K | L | M |  |  |  |  |  |  |
| 1 | 4002 | 4 | 0.364 | 0.025 |  |  |  |  |  |  |  | H | I | J | K | L | M | N |  |  |  |  |  |
| 1 | R09-345 | 3 | 0.364 | 0.025 |  |  |  |  |  |  |  | H | I | J | K | L | M | N |  |  |  |  |  |
| 3 | R09-345 | 3 | 0.362 | 0.021 |  |  |  |  |  |  |  | H | I | J | K | L | M | N |  |  |  |  |  |
| 1 | R09-345 | 2 | 0.358 | 0.025 |  |  |  |  |  |  |  | H | I | J | K | L | M | N |  |  |  |  |  |
| 2 | 8080 | 5 | 0.345 | 0.020 |  |  |  |  |  |  |  |  | I | J | K | L | M | N | O |  |  |  |  |
| 2 | 8080 | 6 | 0.345 | 0.020 |  |  |  |  |  |  |  |  | I | J | K | L | M | N | O |  |  |  |  |
| 1 | R09-345 | 4 | 0.341 | 0.024 |  |  |  |  |  |  |  |  |  | J | K | L | M | N | O |  |  |  |  |
| 1 | 4002 | 5 | 0.339 | 0.024 |  |  |  |  |  |  |  |  |  |  | K | L | M | N | O |  |  |  |  |
| 1 | R09-345 | 5 | 0.335 | 0.024 |  |  |  |  |  |  |  |  |  |  | K | L | M | N | O |  |  |  |  |
| 1 | 8080 | 6 | 0.333 | 0.024 |  |  |  |  |  |  |  |  |  |  | K | L | M | N | O | P |  |  |  |
| 2 | 4002 | 5 | 0.330 | 0.020 |  |  |  |  |  |  |  |  |  |  |  | L | M | N | O | P |  |  |  |
| 3 | 8080 | 5 | 0.327 | 0.020 |  |  |  |  |  |  |  |  |  |  |  | L | M | N | O | P |  |  |  |
| 1 | R09-345 | 6 | 0.317 | 0.032 |  |  |  |  |  |  |  |  |  |  |  | L | M | N | O | P |  |  |  |
| 2 | R09-345 | 4 | 0.310 | 0.018 |  |  |  |  |  |  |  |  |  |  |  | L | M | N | O | P |  |  |  |
| 2 | 4002 | 6 | 0.310 | 0.030 |  |  |  |  |  |  |  |  |  |  |  | L | M | N | O | P | Q |  |  |
| 1 | 4002 | 6 | 0.306 | 0.031 |  |  |  |  |  |  |  |  |  |  |  |  | M | N | O | P | Q |  |  |
| 2 | R09-345 | 6 | 0.299 | 0.030 |  |  |  |  |  |  |  |  |  |  |  |  |  | N | O | P | Q |  |  |
| 3 | 4002 | 4 | 0.298 | 0.022 |  |  |  |  |  |  |  |  |  |  |  |  |  |  | O | P | Q |  |  |
| 2 | R09-345 | 5 | 0.279 | 0.020 |  |  |  |  |  |  |  |  |  |  |  |  |  |  |  | P | Q |  |  |
| 2 | R09-345 | 7 | 0.237 | 0.027 |  |  |  |  |  |  |  |  |  |  |  |  |  |  |  |  | Q | R |  |
| 2 | 4002 | 7 | 0.221 | 0.023 |  |  |  |  |  |  |  |  |  |  |  |  |  |  |  |  |  | R |  |
| 2 | 8080 | 7 | 0.219 | 0.021 |  |  |  |  |  |  |  |  |  |  |  |  |  |  |  |  |  | R |  |
| 3 | R09-345 | 4 | 0.214 | 0.018 |  |  |  |  |  |  |  |  |  |  |  |  |  |  |  |  |  | R |  |
| 3 | 8080 | 6 | 0.173 | 0.025 |  |  |  |  |  |  |  |  |  |  |  |  |  |  |  |  |  | R | S |
| 2 | R09-345 | 8 | 0.156 | 0.022 |  |  |  |  |  |  |  |  |  |  |  |  |  |  |  |  |  |  | S |
| 2 | 4002 | 8 | 0.122 | 0.024 |  |  |  |  |  |  |  |  |  |  |  |  |  |  |  |  |  |  | S |

**Supplemental Table S4.** Least-square mean, Standard Error, and Conservative T-grouping of Edamame Harvest Quality Index (*EHQI*) by cultivar, per planting and harvest date combination, analyzed as a split block design with block and environment as random factors. Levels not connected by same letter are significantly different at α= 0.05.

S4a. R08-4002

| **Planting Date** | **Harvest Date** | **Mean** | **Standard Error** |  |  |  |  |  |  |  |
| --- | --- | --- | --- | --- | --- | --- | --- | --- | --- | --- |
| 3 | 2 | 0.294 | 0.036 | A |  |  |  |  |  |  |
| 2 | 3 | 0.268 | 0.027 | A |  |  |  |  |  |  |
| 3 | 1 | 0.254 | 0.029 | A | B |  |  |  |  |  |
| 3 | 3 | 0.231 | 0.028 | A | B | C |  |  |  |  |
| 2 | 2 | 0.222 | 0.025 | A | B | C |  |  |  |  |
| 2 | 4 | 0.207 | 0.024 |  | B | C | D |  |  |  |
| 2 | 1 | 0.171 | 0.022 |  |  | C | D | E |  |  |
| 2 | 6 | 0.148 | 0.037 |  |  | C | D | E | F |  |
| 1 | 4 | 0.145 | 0.026 |  |  |  | D | E | F |  |
| 1 | 3 | 0.143 | 0.026 |  |  |  | D | E | F |  |
| 1 | 2 | 0.142 | 0.026 |  |  |  | D | E | F |  |
| 2 | 5 | 0.141 | 0.022 |  |  |  |  | E | F |  |
| 1 | 1 | 0.139 | 0.026 |  |  |  |  | E | F |  |
| 1 | 5 | 0.136 | 0.026 |  |  |  |  | E | F |  |
| 1 | 6 | 0.098 | 0.032 |  |  |  |  | E | F | G |
| 3 | 4 | 0.093 | 0.018 |  |  |  |  |  | F | G |
| 2 | 7 | 0.066 | 0.018 |  |  |  |  |  |  | G |
| 2 | 8 | 0.041 | 0.020 |  |  |  |  |  |  | G |

**S4b**. 8080

| **Planting Date** | **Harvest Date** | **Mean** | **Standard Error** |  |  |  |  |  |  |  |
| --- | --- | --- | --- | --- | --- | --- | --- | --- | --- | --- |
| 3 | 3 | 0.309 | 0.033 | A |  |  |  |  |  |  |
| 3 | 1 | 0.297 | 0.033 | A | B |  |  |  |  |  |
| 3 | 2 | 0.250 | 0.030 |  | B | C |  |  |  |  |
| 1 | 4 | 0.210 | 0.031 |  |  | C | D |  |  |  |
| 3 | 4 | 0.207 | 0.027 |  |  | C | D |  |  |  |
| 1 | 3 | 0.195 | 0.029 |  |  | C | D |  |  |  |
| 1 | 5 | 0.194 | 0.029 |  |  | C | D |  |  |  |
| 1 | 1 | 0.179 | 0.028 |  |  |  | D | E |  |  |
| 1 | 2 | 0.164 | 0.027 |  |  |  | D | E | F |  |
| 2 | 2 | 0.163 | 0.023 |  |  |  | D | E | F |  |
| 2 | 3 | 0.161 | 0.022 |  |  |  | D | E | F |  |
| 2 | 4 | 0.156 | 0.022 |  |  |  | D | E | F |  |
| 2 | 6 | 0.139 | 0.020 |  |  |  |  | E | F |  |
| 1 | 6 | 0.133 | 0.023 |  |  |  |  | E | F |  |
| 3 | 5 | 0.130 | 0.020 |  |  |  |  |  | F |  |
| 2 | 5 | 0.128 | 0.019 |  |  |  |  |  | F |  |
| 2 | 1 | 0.127 | 0.019 |  |  |  |  |  | F |  |
| 2 | 7 | 0.053 | 0.012 |  |  |  |  |  |  | G |
| 3 | 6 | 0.033 | 0.010 |  |  |  |  |  |  | G |

**S4c.** R09-345

| **Planting Date** | **Harvest Date** | **Mean** | **Standard Error** |  |  |  |  |
| --- | --- | --- | --- | --- | --- | --- | --- |
| 3 | 2 | 0.158 | 0.019 | A |  |  |  |
| 3 | 3 | 0.144 | 0.016 | A | B |  |  |
| 2 | 3 | 0.144 | 0.014 | A | B |  |  |
| 3 | 1 | 0.142 | 0.016 | A | B |  |  |
| 1 | 3 | 0.132 | 0.018 | A | B |  |  |
| 2 | 2 | 0.127 | 0.013 | A | B |  |  |
| 1 | 4 | 0.126 | 0.017 | A | B | C |  |
| 1 | 5 | 0.125 | 0.017 | A | B | C |  |
| 2 | 1 | 0.121 | 0.013 | A | B | C |  |
| 1 | 6 | 0.113 | 0.023 | A | B | C | D |
| 2 | 4 | 0.112 | 0.012 | A | B | C | D |
| 1 | 1 | 0.112 | 0.016 | A | B | C | D |
| 2 | 6 | 0.109 | 0.022 | A | B | C | D |
| 1 | 2 | 0.107 | 0.016 |  | B | C | D |
| 2 | 5 | 0.092 | 0.012 |  |  | C | D |
| 3 | 4 | 0.071 | 0.011 |  |  |  | D |
| 2 | 7 | 0.062 | 0.013 |  |  |  | D |
| 2 | 8 | 0.060 | 0.016 |  |  |  | D |

**Supplementary Figure S1.** Edamame Harvest Quality Index (*EHQI*) as function of harvest date treatment by planting date treatment for soybean cultivar 8080. Error bars represent standard errors of least-square means for *EHQI*.

**
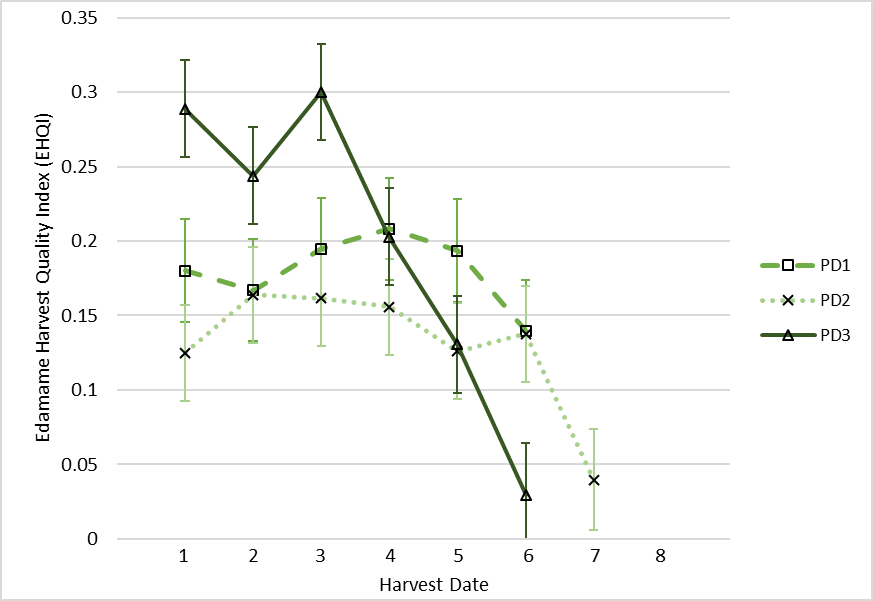
**

**Supplementary Figure S2.** Edamame Harvest Quality Index (*EHQI*) as function of harvest date by planting date for soybean breeding cultivar R08-4002. Error bars represent standard errors of least-square means for *EHQI*.


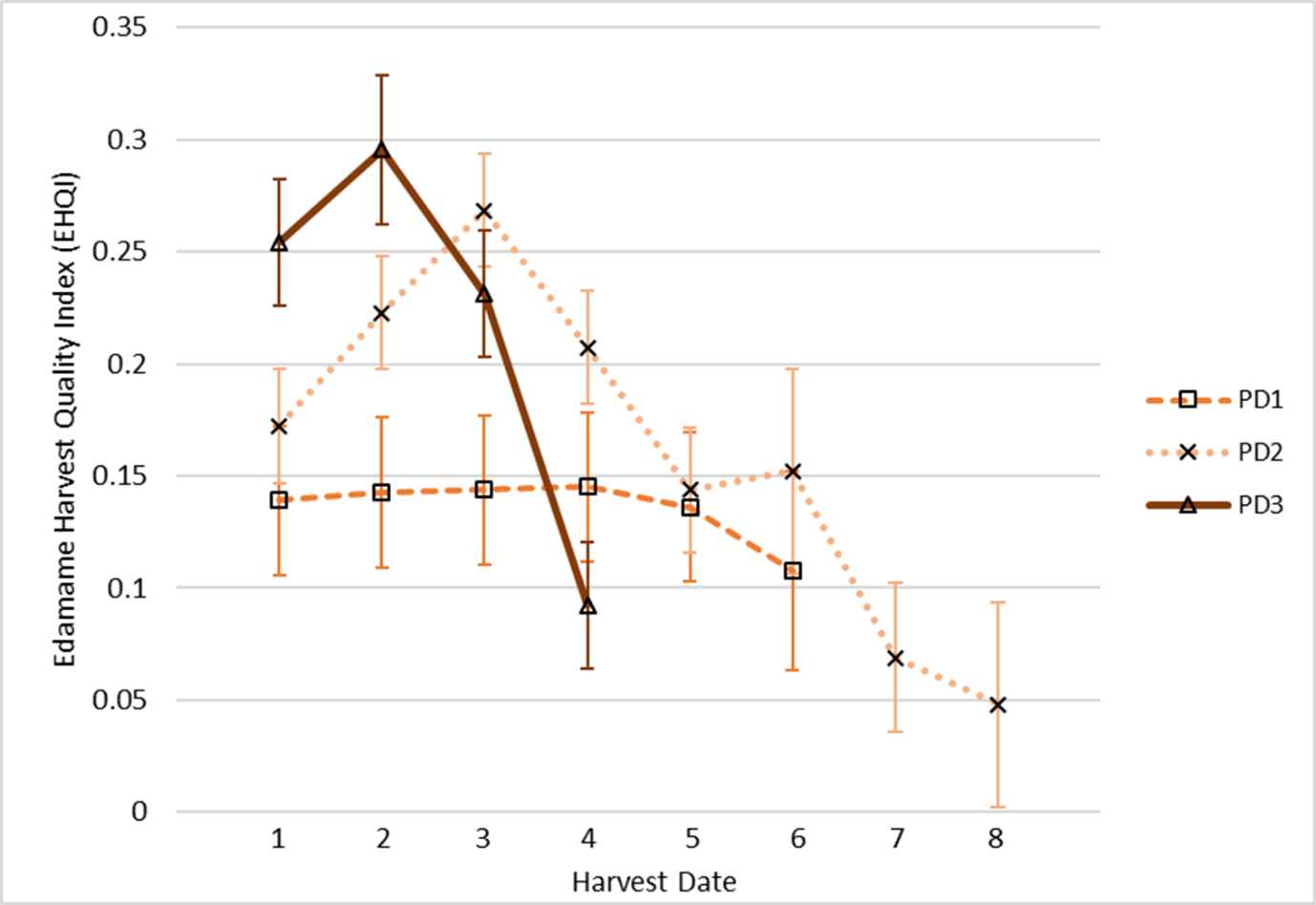


**Supplementary Figure S3.** Edamame Harvest Quality Index (*EHQI*) as function of harvest date by planting date for soybean breeding cultivar R09-345. Error bars represent standard errors of least-square means for *EHQI*.


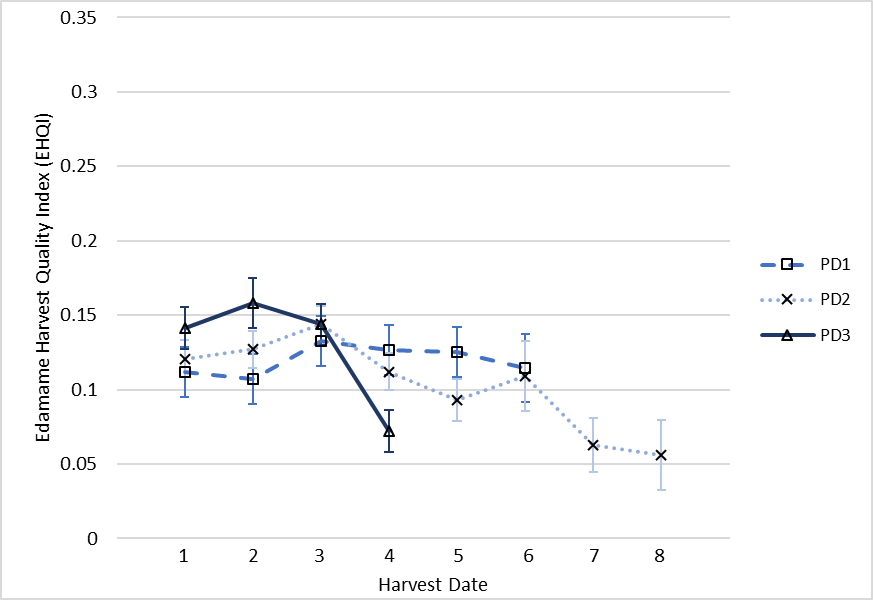

Supplement: Supplementary Figure 1 — Edamame Harvest Quality Index (EHQI) as function of harvest date treatment by planting date treatment for soybean cultivar 8080. Error bars represent standard errors of least-square means for EHQI. [file Data_Sheet_1.docx]
